# Ultrasonographic features of abdominal organs and testicles in captive adult crab-eating foxes (*Cerdocyon thous)* from the Brazilian Cerrado: a case series

**DOI:** 10.1007/s11259-026-11295-0

**Published:** 2026-05-27

**Authors:** Vanessa Martins Fayad Milken, Juliana Moreno Dourado, Mariana Beatriz Rocha Sobrinho, Suzana Akemi Tsuruta, Liliane Rangel Nascimento, André Luis Quagliatto Santos

**Affiliations:** 1 Faculty of Veterinary Medicine and Animal Science, Federal University of Uberlândia, Uberlândia, Brazil; 2 Department of Veterinary Surgery and Animal Reproduction, School of Veterinary Medicine and Animal Science, São Paulo State University, Botucatu, Brazil

**Keywords:** Sonographic features, Abdominal imaging, Wildlife medicine, *Cerdocyon thous*, wild canids

## Abstract

Ultrasonography is a widely used diagnostic imaging technique in veterinary medicine and has gaining ground in wild animal medicine. This case series describes the ultrasonographic anatomy of abdominal organs and testicles in eleven adult captive crab-eating foxes (*Cerdocyon thous*) from the Brazilian Cerrado. All animals were considered clinically healthy based on physical examination and laboratory testing, including complete blood count and serum biochemistry. The animals were sedated with meperidine, acepromazine and propofol prior to ultrasonographic examination. The urinary bladder lumen was anechoic and homogeneous, with a mean wall thickness of 0.17 cm. The prostate was visualized caudal to the bladder, with mean length and height of 1.61 cm and 1.21 cm, respectively. The spleen showed homogeneous hyperechoic parenchyma with a mean thickness of 1.20 cm. Kidneys demonstrated well-defined corticomedullary differentiation, with mean lengths of 4.08 cm (left) and 4.15 cm (right). The liver displayed smooth margins, uniform echotexture, and intermediate echogenicity relative to the spleen and renal cortex. The gallbladder appeared as an anechoic structure with a mean wall thickness of 0.10 cm. The stomach wall measured 0.24 cm, intestinal wall thickness averaged 0.35 cm in the small intestine and 0.15 cm in the large intestine. Testicles displayed an oval shape with homogeneous echotexture and a visible mediastinum testis. Overall, the sonographic features observed were consistent with those reported in domestic dogs. These results aid to establishing reference ultrasonographic parameters for *Cerdocyon thous* and may facilitate clinical evaluation and diagnostic imaging in this species.

## Background

Clinical management of wild animals is frequently difficult due to the absence of well-defined physiological, morphological, and anatomical parameters, as well as the lack of clinical history in free-living individuals. Establishing anatomical reference standards is essential for expanding knowledge throughout diverse wildlife species and supporting clinical decision-making in veterinary practice (Cooper and Cooper 2006).

Abdominal ultrasonography is a valuable diagnostic tool due to its safety, noninvasive nature, and capacity for real-time visualization. Furthermore, it can be conducted in field settings during emergencies (Gardhouse 2023). Ultrasonography also has an important role in research and conservation initiatives by supporting reproductive management and health monitoring of endangered species (Cubas et al. 2014; Mackey et al. 2008). When dealing with wild animals, the use of ultrasonography presents some challenges, as there is limited data on these species, making interpretation of exams more difficult in determining what is normal or abnormal in their ultrasonographic anatomy (Redrobe 2001).

The crab-eating fox (*Cerdocyon thous*) is a neotropical canid (Beisiegel et al. 2013; De Thoisy et al. 2013) with considerable ecological plasticity, allowing it to prosper in anthropized landscapes (Hody et al. 2019; Santos et al. 2024). However, habitat fragmentation and increased human activity have aggravated interactions with urban environments, leading to a higher frequency of anthropogenic injuries, particularly road mortality (Pinto et al. 2022). Consequently, this species is more frequently encountered in wildlife centers, where clinical and diagnostic evaluation becomes essential for screening, identifying conditions that may preclude release, and guiding decisions regarding rehabilitation or management (Ichikawa et al. 2025).

There is only one study that describes the ultrasonographic findings in *Cerdocyon thous* (Silva et al. 2014), based on a sample of young individuals (8 months to 1 year of age), representing an important reference for juvenile animals. However, considering that ultrasonographic characteristics may vary according to age, information on adult individuals remains unexplored. While the available data suggests general similarities in findings, the inclusion of animals from different age groups contributes to a broader understanding of the species.

This report describes the ultrasonographic findings of major abdominal organs and testicles in 11 captive adult crab-eating foxes, one of the principal canid species in Brazil, in order to establish reference data of the normal ultrasonographic appearance. Such information may facilitate clinical management of these animals under human care and improve the diagnostic accuracy of imaging examinations.

## Case presentation

Eleven young adult captive Crab-eating foxes (*Cerdocyon thous*), including eight males and three females, were allocated in the Teaching and Research Laboratory for Wild Animals (LAPAS), in the Federal University of Uberlandia (UFU), Brazil. The animals were rescued by the Environmental Police in the Triângulo Mineiro, including orphaned juveniles and animals found in urban areas, and were subsequently maintained under veterinary care at the present institution until reaching maturity and being deemed suitable for release and destination, for at least 1 year. All animals underwent clinical screening, including physical examination, hematological and biochemical analyses. Only individuals considered clinically healthy under these evaluations were included in the study. Body weight ranged from 5.2 to 7.4 kg.

Prior to the ultrasonographic examination, the animals were fasted for 8 h and water-restricted for 4 h. They were physically restrained with nets for the application of pre-anesthesia medication by intramuscular administration of meperidine (4 mg/kg) and acepromazine (0,05 mg/kg). The maintenance was made by intravenous administration of propofol (5 mg/Kg). During the procedure, heart rate, respiratory rate, and mucosal color were monitored. Chemical restraint was necessary to minimize stress and ensure the safety of both the animals and the veterinary team. No complications were observed during anesthesia or recovery.

Ultrasound examination was performed using ultrasound equipment (Logic F6; GE HealthCare) with a microconvex (4-10Mhz) and linear (6-12Mhz) transducers. The animals were positioned in dorsal recumbency, and ventral abdominal fur was clipped, along with the use of an acoustic coupling gel spread over the ventral abdomen for the scan.

Bidimensional images were obtained in both longitudinal and transverse planes to evaluate the urinary bladder, prostate, kidneys, spleen, liver, gallbladder, stomach, intestines, and testicles. All data were collected by the same experienced investigator. Images were digitally stored and later systematically evaluated regarding organ topography, form, margins, dimensions, thickness, echogenicity, and echotexture. The positioning and sequence of the exam were maintained for all animals to standardize the study. The values obtained with the measurement of the organs were systematically organized and processed to determine the values of descriptive statistics (mean and standard deviation).

**Fig. 1:**
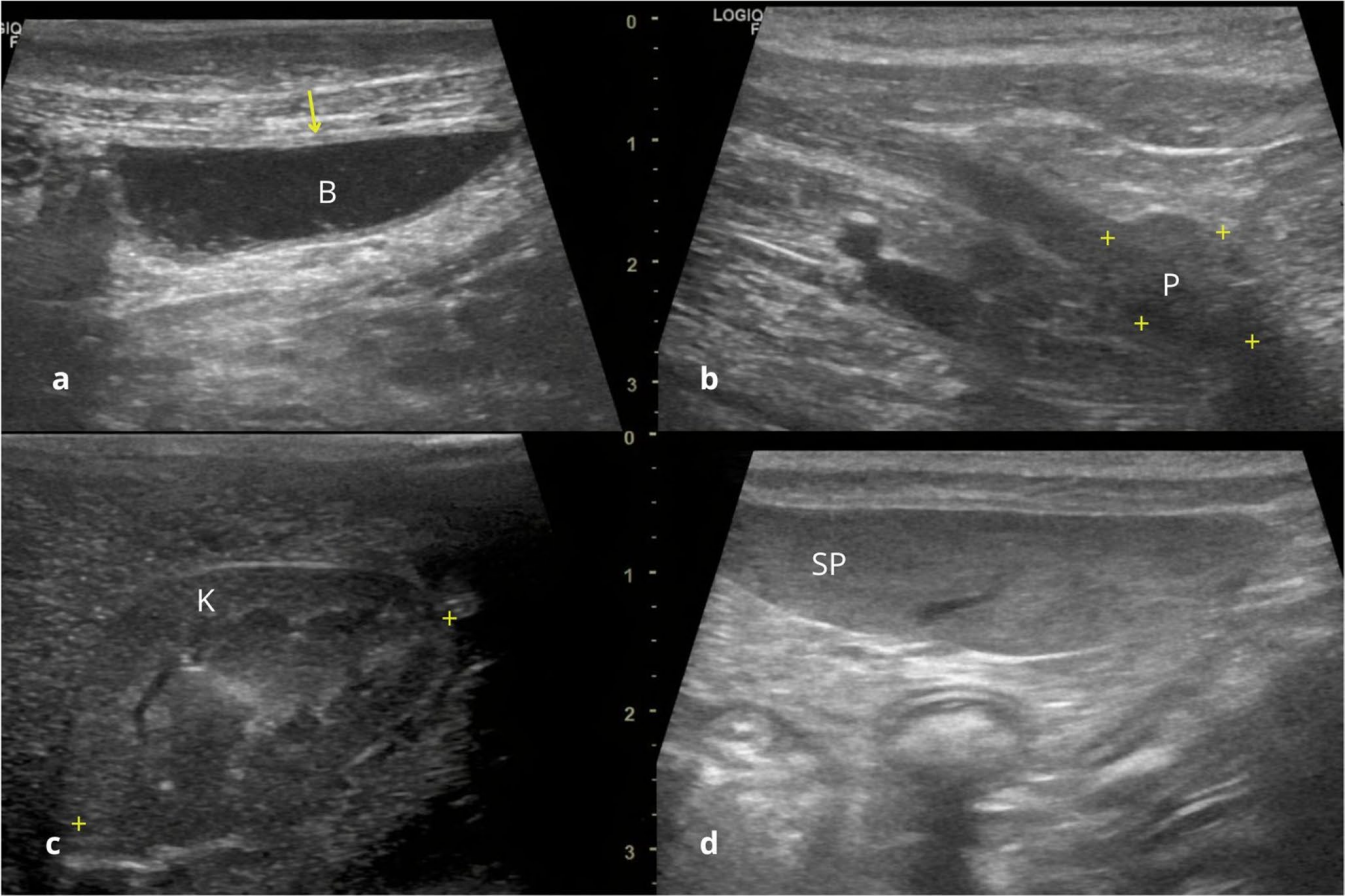
Ultrasonographic images of healthy adult crab-eating fox. (**a**) Moderately distended urinary bladder (B) with anechoic content. Note the three layers (arrow). (**b**) Prostate (P) showing rounded shape, hypoechoic and homogeneous echotexture (P). (**c**) Right kidney (K) with clear corticomedullary differentiation and echogenicity slightly lower than that of the spleen. (**d**) Triangular-shaped spleen (SP) with hyperechoic parenchyma and homogeneous echotexture

The urinary bladder (Fig. 1-a) was in the caudal abdomen, cranial to the genital opening and ventral to the large intestine. In six animals, the bladder was not fully distended, which may be attributed to urine voiding during restraint and to water restriction prior to the exam. The content was homogeneous and anechoic. The bladder was examined in both longitudinal and transverse planes, and the mean thickness was 0,17 cm (+/- 0,07 cm – Table 1).

**Table 1:** Descriptive statistics of ultrasonographic measurements (cm) of the main abdominal organs and testicles of healthy adult crab-eating foxes (*Cerdocyon thous*)

Organ (*N*)	Measurement	Mean	SD	Min	Max
Urinary bladder (11/11)	Wall thickness	0.17	0.06	0.1	0.36
Prostate (2/8)	Length	1.61	0.07	1.54	1.68
	Width	1.21	0.28	0.93	1.49
Right kidney (11/11)	Length	4.15	0.28	3.75	4.83
	Width	2.23	0.21	1.88	2.54
Left kidney (11/11)	Length	4.08	0.32	3.37	4.63
	Width	2.12	0.40	1.74	2.43
Spleen (11/11)	Thickness	1.20	0.33	0.9	2.15
Gallbladder (11/11)	Wall thickness	0.10	0.03	0.06	0.18
Stomach (11/11)	Wall thickness	0.24	0.07	0.13	0.34
Right testicle (7/8)	Length	1.83	0.24	1.53	2.2
	Width	0.94	0.10	0.79	1.07
Left testicle (7/8)	Length	1.77	0.30	1.36	2.28
	Width	0.82	0.15	0.65	1.03

*N* = number of animals in which the organ was visualized / total number of animals included

*SD *standard deviation, *Min* minimum, *Max* maximum

The prostate (Fig. 1-b) was visualized in only two males (2/8), positioned caudal to the urinary bladder. The limited visualization of the prostate may be attributed to reduced urinary bladder distension, resulting in a predominantly intrapelvic position, probably due to spontaneous urination during physical restraint in several individuals. Its margins were well defined, and the parenchyma displayed a homogenous echotexture with hypoechoic echogenicity relative to surrounding tissues. The prostatic urethra was visible in the center as a thin anechoic structure, in transverse plane. Prostatic length and height ranged from 0,93 to 1,49 cm and 1,54 to 1,68 cm, respectively, with standard deviation of ± 0.28 cm and ± 0.07 cm (Table 1).

Both kidneys were consistently visualized. The right kidney (Fig. 1-c) was located cranially in relation to the contralateral, in the renal fossa of the right hepatic lobe, under the costal arch, while the left was observed medially to the spleen. The kidneys presented a well-defined hyperechoic capsule and a homogenous echotexture. Renal echogenicity was lower when compared to the spleen and similar to, or slightly less than, that of the liver. The cortical and medullary regions were clearly distinguishable (ratio 1:1), with the medulla appearing less echogenic and the cortex more echogenic. The maximum length of the kidney in longitudinal plane 4,83 cm to the right and 4,63 cm to the left (Table 1).

The spleen (Fig. 1-d) was visualized in the left cranial abdomen, with a dorsoventral orientation. The organ exhibited a triangular shape and was surrounded by a thin, smooth, echogenic and regular capsule. Located caudal to the liver and lateral to the stomach, the splenic body and tail extended throughout the left abdominal region. The parenchyma displayed a homogenous hyperechoic when compared to the liver and renal cortex, assessed by imaging these structures within the same plane. The dimensions were assessed subjectively using criteria established for domestic dogs, such as the presence of building margins, showing slight splenomegaly in most of the animals. The mean thickness of the spleen was 1,20 cm (Table 1).

**Fig. 2:**
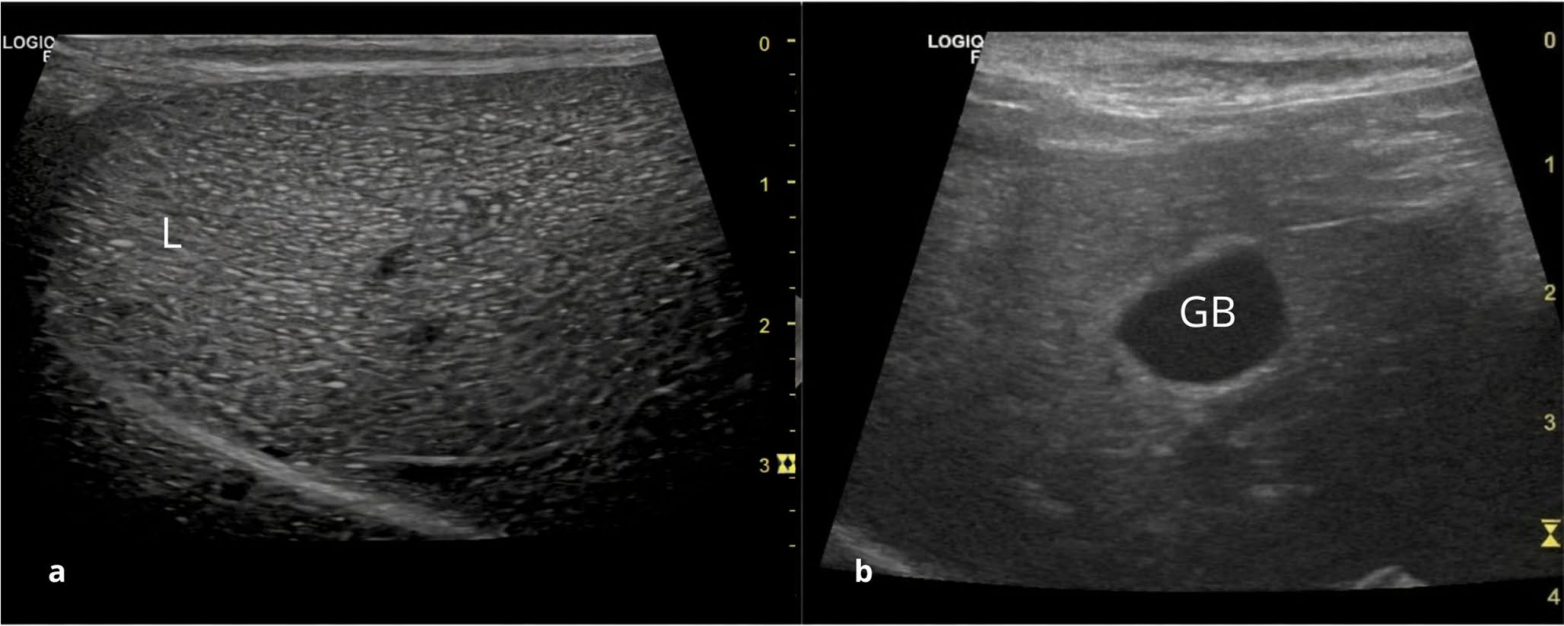
Ultrasonographic images of the liver (L) and gallbladder (GB) in healthy adult crab-eating fox. (**a**) Hepatic parenchyma with homogeneous echotexture. (**b**) gallbladder with homogeneous anechoic content

The liver (Fig. 2-a) was evaluated by positioning the transducer caudal to the xiphoid process and directing it craniodorsally, allowing visualization of the diaphragm as a hyperechogenic line. The beam was swept in an arc to obtain a complete scan of the organ. The liver exhibited smooth margins and a uniform echotexture, slightly coarser than that of the spleen, with intermediate echogenicity relative to the spleen and renal cortex. Several blood vessels of varying calibers were evident within the parenchyma, appearing as tubular anechoic structures, portal branches were particularly prominent due to their echogenic walls, which facilitated their identification. Liver size assessment was subjective, the parameters used to evaluate alterations were based on those established for domestic dogs. No hepatomegaly was observed the examined animals.

The gallbladder (Fig. 2-b) has a piriform shape and may present with variable size on ultrasound, depending on the volume of bile stored in the moment of the exam. The content was homogeneous and anechoic. It is located between the right lobe and quadrate lobe, and its wall is very thin, appearing as an echogenic line. The gallbladder was examined in both longitudinal and transverse planes, and the mean thickness was 0,10 cm (Table 1).

**Fig. 3:**
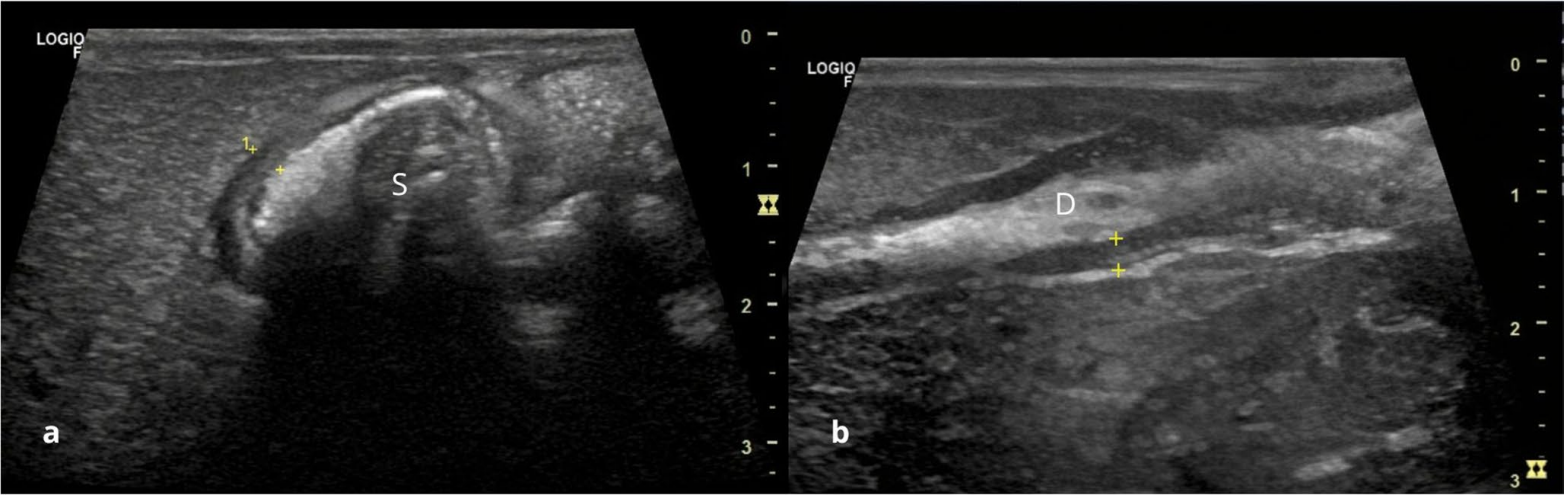
Ultrasonographic images of part of the gastrointestinal tract in healthy adult crab-eating fox, showing the typical layered appearance of: **a **– stomach (S); **b **– duodenum (D). The intestinal wall stratification is visible, consisting of the mucosa (hypoechoic), submucosa (hyperechoic), muscular layer (hypoechoic), and serosa (hyperechoic), from the lumen outward (between calipers)

The stomach (Fig. 3-a) was visualized in the cranial abdomen, positioned caudal to the liver, craniomedial to the spleen, and cranial to the left kidney. It was characterized by the presence of mucosal folds and regular peristaltic movements. The gastric wall had a mean thickness of 0.24 cm (± 0,07 cm – Table 1), measured from the outer hyperechoic serosal layer to the inner hyperechoic interface representing the mucosal surface. Due to the fasting period prior to the examination, the gastric lumen did not contain food material. Although artifacts caused by gas within the gastrointestinal tract were minimized, a small amount of intraluminal gas was still observed. The small intestine (Fig. 3-b) was diffusely distributed within the abdominal cavity, caudal to the stomach and liver, with a mean wall thickness of 0.36 cm. The large intestine, organized into ascending, transverse, and descending colon, extended from the right cranial to the left caudal abdomen, with a mean wall thickness of 0.15 cm. The five intestinal wall layers (serosa, muscularis, submucosa, mucosa and lumen) were clearly distinguishable during ultrasonographic examination.

**Fig. 4:**
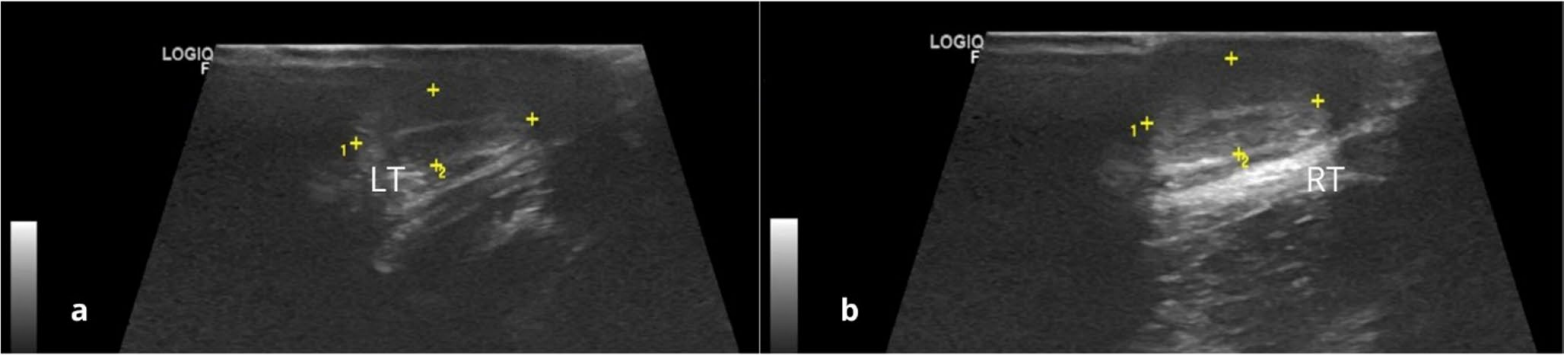
Ultrasonographic images of the testicles showing a central hyperechoic line in healthy adult crab-eating fox. LT = left testicle (**a**); RT = right testicle (**b**)

Of the eleven animals evaluated, eight were males and underwent ultrasonographic examination of the testicles (Fig. 4). Located within the scrotum, the testes exhibited an oval shape, and measurements were obtained in both longitudinal and transverse planes. The testicular mediastinum appeared as a hyperechoic central structure relative to the surrounding testicular parenchyma, which displayed homogeneous echotexture. The right testicle showed larger dimensions than the left, both in length and width, with measurements ranging from 1.36 to 2.28 cm in length and 0.65 to 1.03 cm in width (Table 1).

## Discussion and conclusions

There are relatively few studies describing the ultrasonographic anatomy of crab-eating foxes and other Brazilian wild canids (Guimarães et al. 2013; Silva et al. 2014). In the present case series, the location and characteristics of the analyzed organs showed strong similarity to those described for domestic dogs. This correspondence indicates that imaging methods established for companion animals may serve as a reliable foundation for the ultrasonographic evaluation of this wild canid species.

A fully distended urinary bladder serves as an acoustic window that facilitates the sequential assessment of adjacent structures (Mattoon et al. 2021; Penninck and D’Anjou 2025). However, in the clinical routine for wild animals, it is difficult to ensure a completely fully urinary bladder due to stress during handling and consequent spontaneous urination. The bladder exhibits a three-layered wall similar to that described for other mammals (Barreto et al. 2021; Augusti et al. 2023; Barkman et al. 2023). The mean thickness obtained (0,17 cm) aligned with values observed in domestic canids of equivalent size (Mattoon et al. 2021; Penninck and D’Anjou 2025) and lower (0,17 to 0,28 cm) and slightly higher (0,12) than that reported for maned wolves (*Chrysocyon brachyurus*) and young crab-eating foxes for Guimarães et al. (2013) and Silva et al. (2014), respectively. As described in dogs, bladder wall thickness is influenced by the degree of distention (Penninck and D’Anjou 2025), and this factor must be considered when interpreting measurements in wild animals whose bladder filling cannot always be standardized.

Prostatic echogenicity and echotexture were inconsistent with descriptions for domestic dogs (Penninck and D’Anjou 2025) and crab-eating foxes (Silva et al. 2014) for the echogenicity, however consistent with the observed by Guimarães et al. (2013) for maned wolf, where the authors had limited visualization of the prostate due to similarity of echogenicity to adjacent tissues. Nevertheless, the gland was visible in only two of the eight males, probably due to insufficient bladder distention, which made it more difficult to locate the gland. As in domestic dogs, size variations may also be influenced by individual and species-specific factors.

Renal evaluation revealed well-defined corticomedullary differentiation, with cortical echogenicity consistently lower than that of the spleen and similar or slightly inferior to that of the liver, as reported in domestic dogs (Penninck and D’Anjou 2025) and crab-eating foxes (Silva et al. 2014). In contrast, Guimarães et al. (2013) described that the liver and kidneys have the same echogenicity in all evaluated animals. The mean kidney length matched values reported in other canids (Silva et al. 2014; Mattoon et al. 2021). However, in maned wolves, Guimarães et al. (2013) found higher renal dimensions, ranging from 6,55 cm to 8 cm, which may be due to the animals in the study weighing between 19.3 and 28 kg. As in other canids, the right kidney tended to present slightly larger measurements than the left.

The spleen was consistently visualized in the cranial left abdomen and exhibited a homogeneous and hyperechoic parenchyma that is aligned with the description for domestic dogs (Mattoon et al. 2021; Penninck and D’Anjou 2025). In contrast, Guimarães et al. (2013) reported a small difference in the maned wolf, with most animals showing very similar echogenicity between the spleen and renal cortex. Given the observed thickness, the acepromazine included in the anesthetic protocol may have contributed to increased splenic size in some individuals by relaxing the smooth muscles of the capsule, thereby increasing the organ’s dimensions through greater blood storage (Sutil et al. 2017). Therefore, splenic measurements obtained under anesthesia should be interpreted with caution.

The anatomical position and ultrasonographic appearance of the stomach were consistent with those described for domestic dogs and others wild canids (Guimarães et al. 2013; Silva et al. 2014; Mattoon et al. 2021; Penninck and D’Anjou 2025). Gas within the gastrointestinal tract may be observed in both fasted and unprepared animals (Garcia and Froes 2014). In the present study, fasting was primarily implemented to ensure safe administration of anesthetic drugs. Although antiflatulent medication was not administered due to difficulty administering oral medication in these animals, the small amount of gas observed did not compromise the interpretation of the ultrasonographic images.

The small intestine (duodenum and jejunum) had a mean wall thickness of 0,37 cm, comparable to values reported for domestic dogs weighing less than 20 kg (Mattoon et al. 2021) and slightly less than the observed for young crab-eating foxes (Silva et al. 2014). Layer definition and echogenicity also resembled those described for other wild canids (Guimarães et al. 2013; Silva et al. 2014), showing a high degree of anatomical consistency among these species.

The hepatic parenchyma showed a pattern of echogenicity similar to that observed in other mammals (Barreto et al. 2021; Augusti et al. 2023; Barkman et al. 2023), with an intermediate echogenicity between the spleen and renal cortex. In maned wolves, however, the hepatic parenchyma has been described as having a coarser texture and echogenicity similar to the renal cortex (Guimarães et al. 2013).

The gallbladder was moderately full in all animals and located to the right of the midline, resembling the positioning described in dogs (Mattoon et al. 2021; Penninck and D’Anjou 2025). Wall thickness values in this study (0.10 cm) were aligned with those reported for domestic dogs by Martinez et al. (2023). Comparison with other wild canids was not possible due to the absence of gallbladder wall measurements in previously published studies.

Testicular ultrasonographic features, including echogenicity, visualization of the mediastinum, and parenchymal appearance, were similar to those described in domestic dogs (Mattoon et al. 2021) and differed from the maned wolf, which presented a hypoechogenic testicular parenchyma with coarse echotexture and poorly defined mediastinum (Guimarães et al. 2013).

Overall, the ultrasonographic protocol commonly used in domestic dogs proved applicable for evaluating captive crab-eating foxes. The ultrasonographic anatomy observed in this species appeared more similar to that of domestic dogs than to that reported in maned wolves, another Brazilian wild canid species, described by Guimarães et al. (2013).

When compared with the only previously published article about ultrasonography in *Cerdocyon thous* (Silva et al. 2014), the findings of the present study showed overall agreement regarding organ echogenicity, parenchymal echotexture, and general anatomical distribution. However, some differences were observed relative to the measurements obtained, which may be related to variations in sample size, animal condition, and examination protocols, and for prostatic echogenicity. Additionally, age differences between study populations should be considered, as previous studies evaluated younger animals (8 months to 1 year of age), whereas the present study describes only adult individuals. These factors may contribute to variations in ultrasonographic parameters within the same species.

From a clinical perspective, the reference ultrasonographic parameters described in this study may help distinguish normal anatomical findings from pathological conditions in adult individuals of *Cerdocyon thous*. Given the anatomical and echogenic similarities observed between these species and domestic dogs, variations from the patterns reported here may serve as important indicators of disease and support diagnosis. These baseline data are particularly relevant in wildlife clinical settings, where limited species-specific information often hampers diagnostic interpretation. In addition, they may assist veterinarians who are not routinely involved in wildlife practice but may occasionally be required to perform diagnostic imaging in these animals, providing a useful reference not only for normal measurements but also as a visual framework for comparison.

However, some limitations should be considered when interpreting the findings. The sample size was relatively small (*n* = 11). Additionally, constraints related to wildlife handling, including the demand for chemical restraint, may limit the duration of the examination and, consequently, the comprehensive assessment of all organs. This was especially evident in the evaluation of the prostate, which was visualized in only two individuals, likely due to factors such as insufficient urinary bladder distension. Therefore, ultrasonographic evaluation in these animals requires both agility and operator experience to obtain reliable data within a restricted timeframe. Given the scarcity of ultrasonographic data on Brazilian wild canids, further studies involving larger populations and additional species are necessary to consolidate baseline parameters and increase diagnostic accuracy in wildlife medicine.

In conclusion, this case series provides descriptive ultrasonographic information regarding the main abdominal and genital organs of captive adult crab-eating foxes. This evidence may serve as a useful reference for clinical evaluation and diagnostic imaging in this species, aiding the identification of pathological conditions through a non-invasive approach and supporting veterinary care and conservation initiatives for wild canids.

## Data Availability

The data will be available from the corresponding author upon request.
